# Correction: Suriya et al. Integration of In Silico Strategies for Drug Repositioning towards P38α Mitogen-Activated Protein Kinase (MAPK) at the Allosteric Site. *Pharmaceutics* 2022, *14*, 1461

**DOI:** 10.3390/pharmaceutics17040419

**Published:** 2025-03-26

**Authors:** Utid Suriya, Panupong Mahalapbutr, Thanyada Rungrotmongkol

**Affiliations:** 1 Program in Biotechnology, Faculty of Science, Chulalongkorn University, Bangkok 10330, Thailand; 2 Department of Biochemistry, Center for Translational Medicine, Faculty of Medicine, Khon Kaen University, Khan Kaen 40002, Thailand; 3 Center of Excellence in Structural and Computational Biology, Department of Biochemistry, Chulalongkorn University, Bangkok 10330, Thailand; 4 Ph.D. Program in Bioinformatics and Computational Biology, Graduate School, Chulalongkorn University, Bangkok 10330, Thailand

## Error in Figure and Table

In the original publication [1], there was a mistake in the structure of Nilotinib; the oxygen at the amide group was changed to an -OH group. It occurred from the post-docking step and was used as an initial structure for MD simulations. So, we re-performed the calculations on Nilotinib and corrected the figures to show the new results of Nilotinib. We corrected the 2D chemical structures, binding energy of ZINC6716957 (Nilotinib), intermolecular interactions of ZINC6716957 (Nilotinib), percentage of H-bond occurrence of Nilotinib, per-residue free energy decomposition of Nilotinib, and ΔG_bind_ values of ZINC6716957 (Nilotinib). The corrected items, Figure 2, Figure 3, Figure 4, Figure 5, Figure 6, Figure S3 and Figure S4 and Table 1 and Table S2, appear below.

## Text Correction

The results from MD simulations of Nilotinib were re-calculated since its structure was corrected. A correction has been made to the ΔG_bind_, ΔG_RF_, and non-polar solvation effect of Nilotinib in Section 3.2. Dynamic-Based Screening and End-Point Binding Free Energy Calculations, paragraph 2:

The sentence “For screening purposes, only two hit compounds, lomitapide (ΔG_bind_ = −11.39 ± 0.05 kcal/mol) and nilotinib (ΔG_bind_ = −11.27 ± 0.03 kcal/mol) displaying a similar level of binding strength to the BIRB796, were selected for further investigation” was added in Chapter 3.2, Page 7, Line 252–254.

The sentence “Lomitapide and nilotinib showed a slightly higher ΔG_RF_ (17.01 ± 0.24 and 17.15 ± 0.17, respectively) than BIRB796 (15.60 ± 0.20), implying the relatively minute higher polar solvation in the lomitapide and nilotinib complex system” was added in Chapter 3.2, Page 8, Line 267–269.

The sentence “while nilotinib and BIRB796 demonstrated a slight reduction in the nonpolar solvation effect (−12.81 ± 0.04 and −13.63 ± 0.04, respectively)” was added in Chapter 3.2, Page 8, Line 272–274.

A correction has been made to Section 3.3. Contact Atoms and Numbers of Hydrogen Bond Formation, paragraph 1–3:

The phrase “nilotinib (396 ± 19 atoms)” was added in Chapter 3.3, Page 8, Line 289.

The sentence “the numbers of averaged H-bond interactions in BIRB796 were approximately 4 bonds while lomitapide and nilotinib were in a vicinity of 2 and 3 bonds, respectively” was added in Chapter 3.3, Page 9, Line 295–297.

The phrase “97.2% occupations” was added in Chapter 3.3, Page 9, Line 309.

The sentence “the BIRB796 and nilotinib could form an additional very strong H-bond with E71 (2 H-bonds for BIRB796 and 1 H-bond for nilotinib)” was added in Chapter 3.3, Page 9, Line 310–312.

A correction has been made to Section 3.4. Key Binding Residues, paragraph 2: 

The phrase “I84” was added in Chapter 3.4, Page 10, Line 348.

The sentence “In the case of nilotinib, it was found that key amino acids contributing to its binding were mostly the same residues responsible for BIRB796 binding (E71, I84, L167, and D168)” was added in Chapter 3.4, Page 11, Line 356–358.

The sentence “Two additional residues, L74 and M109 were also observed” was added in Chapter 3.4, Page 11, Line 361.

The authors state that the scientific conclusions are unaffected. This correction was approved by the Academic Editor. The original publication has also been updated.

## Figures and Tables

**Figure 2:**
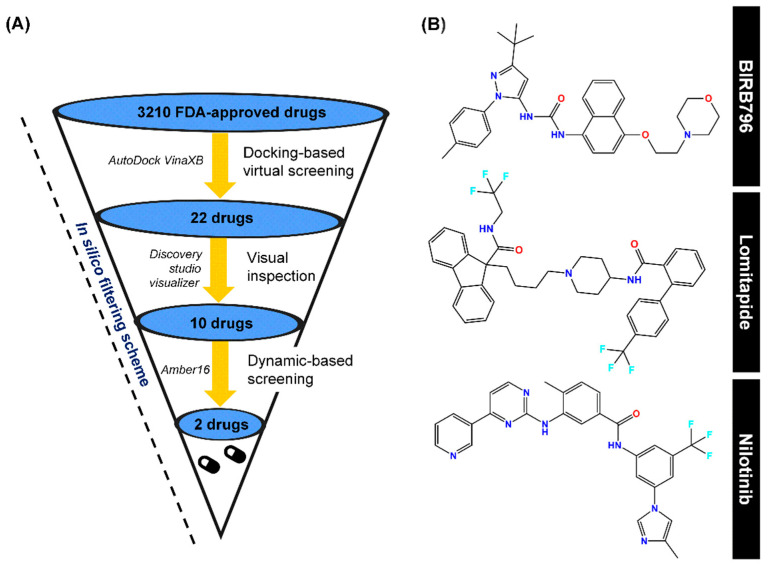
(**A**) In silico filtering scheme, which includes first-round docking-based screening, visual inspection, and SIE-based dynamic screening as well as the program used during each step. (**B**) Chemical structures of a well-known p38α MAPK allosteric inhibitor (BIRB796), Lomitapide, and Nilotinib, obtained via this computational platform.

**Figure 3:**
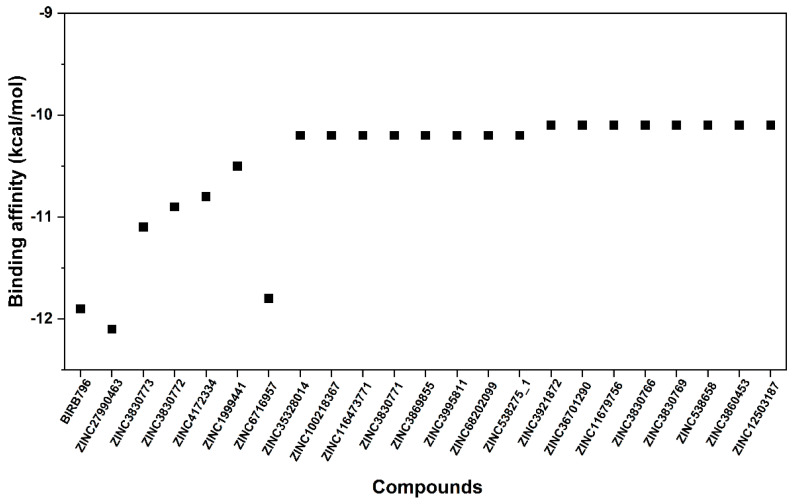
Binding affinity in kcal/mol of selected first-round screened compounds that were successfully docked into the focused allosteric site of p38α MAPK compared to BIRB796. Note that the prediction was based upon the scoring function implemented in the Autodock VinaXB, Sirimulla Research Group at the University of Texas at El Paso, TX, USA.

**Figure 4:**
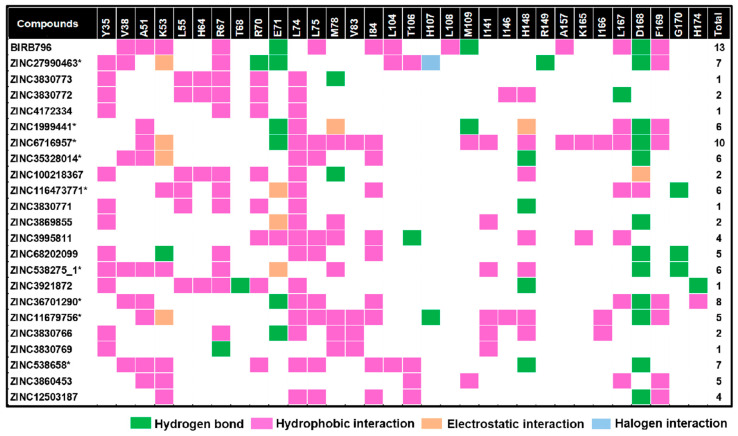
Map of intermolecular interactions of BIRB796 and all 22 screened compounds as well as total features sharing interactions with BIRB796. Each type of noncovalent interaction was also illustrated in different colors. It is worth noting that these occurred interactions were based upon the best docked conformation and visualized by Accelrys Discovery Studio 2.5. * The compounds selected to run MD simulations.

**Figure 5:**
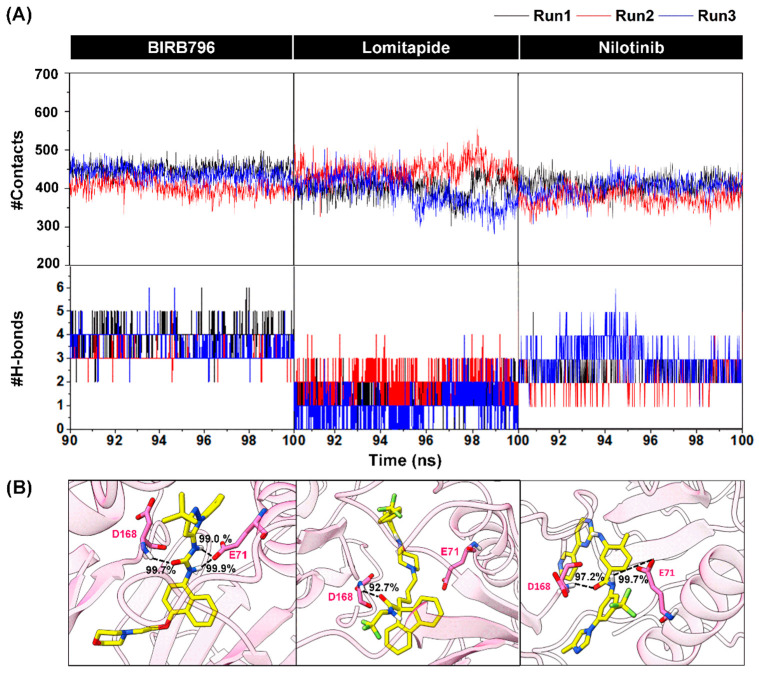
(**A**) Numbers of surrounding atoms counted within the 5.0 Å from the ligand and number of H-bonds within p38α MAPK-BIRB796 complex and two focused drugs at the last 10 nanoseconds (90–100 ns). The results were shown in three independent runs. (**B**) Percentage of H-bond occurrence during a complex formation of two screened drugs and the BIRB796 using two criteria as follows: (1) the distance between the hydrogen bond donor (HD) and hydrogen acceptor (HA) of ≤3.5 Å (2) the angle ≥120°.

**Figure 6:**
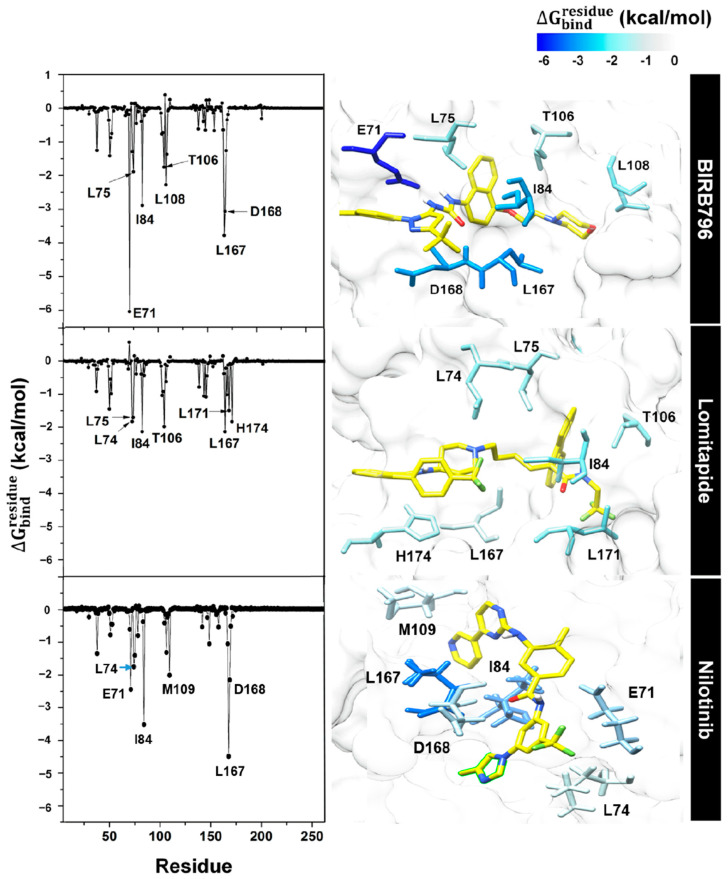
Per-residue free energy decomposition of amino acids involved in ligand binding where the highest to lowest ${\Delta G}_{residue}^{bind}$ contribution (more negative value) was shaded from dark blue to white.

**Figure S3:**
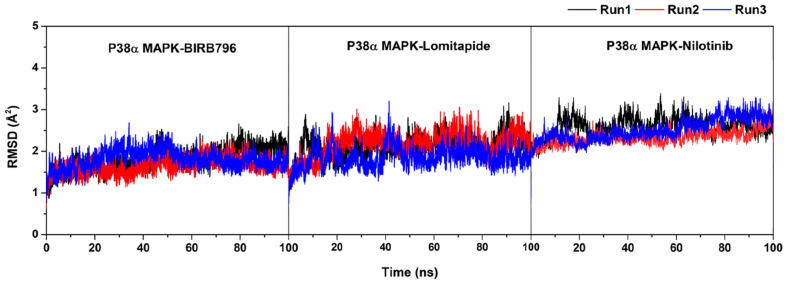
Plot of root-mean-square displacement (RMSD) for the protein-ligand complexes. The data were illustrated in three independent runs with different initial velocities.

**Figure S4:**
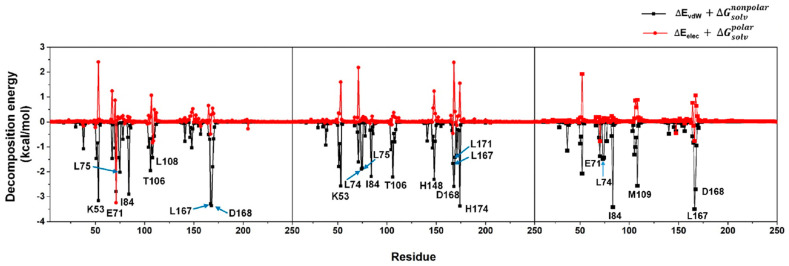
Analysis of per-residue VdW and electrostatic decomposition energy in which the amino acids largely contributed via VdW and electrostatic interaction energies were labelled as a one-letter code format. We noted that even though some residues (e.g., K53, H148) were noticeably stabilized through vdW interactions, they did not play a role in the binding process since they were destabilized by electrostatic charge-charge repulsion (as observed in a positive electrostatic energy). Hence, these kinds of residues were not shown in the article Figure 6.

**Table 1:** ΔG_bind_ values (kcal/mol) of the candidate compounds as well as BIRB796 in complex with p38α MAPK calculated by the SIE-based end-point method using α, γ, and constant coefficients of 0.10, 0.01, and −2.89, respectively.

Drugs (ZINC ID)	Energy Components (kcal/mol)				
	E_VdW_	E_coul_	ΔG_RF_	ΔG_cavity_	ΔG_bind_
BIRB796	*experiment*				−10.98 *
	−78.53 ± 0.29	−9.93 ± 0.15	15.60 ± 0.20	−13.63 ± 0.04	−11.95 ± 0.04
Lomitapide (ZINC27990463)	−77.77 ± 0.39	−5.95 ± 0.18	17.01 ± 0.24	−14.43 ± 0.04	−11.39 ± 0.05
Nebivolol (ZINC1999441)	−49.57 ± 0.29	−12.66 ± 0.26	12.78 ± 0.21	−10.19 ± 0.03	−9.14 ± 0.03
Nilotinib (ZINC6716957)	−71.93 ± 0.26	−12.36 ± 0.19	17.15 ± 0.17	−12.81 ± 0.04	−11.27 ± 0.03
Ibrutinib (ZINC35328014)	−61.87 ± 0.29	−4.07 ± 0.17	13.21 ± 0.23	−10.81 ± 0.04	−9.55 ± 0.04
Atovaquone (ZINC116473771)	−42.15 ± 0.27	−2.43 ± 0.39	10.94 ± 0.23	−7.90 ± 0.05	−7.24 ± 0.03
Dicumarol (ZINC3869855)	−39.68 ± 0.26	−13.87 ± 0.40	20.55 ± 0.32	−7.13 ± 0.03	−7.09 ± 0.03
Raloxifene (ZINC538275)	−51.19 ± 0.44	−12.45 ± 0.50	18.50 ± 0.25	−9.90 ± 0.05	−8.66 ± 0.07
Ponatinib (ZINC36701290)	−61.66 ± 0.25	−4.92 ± 0.14	16.99 ± 0.24	−12.08 ± 0.05	−9.35 ± 0.03
Eltrombopag (ZINC11679756)	−59.44 ± 0.33	−5.39 ± 0.17	10.52 ± 0.17	−10.97 ± 0.03	−9.73 ± 0.04
Samsca (ZINC538658)	−51.69 ± 0.26	−16.63 ± 0.21	19.86 ± 0.18	−10.38 ± 0.04	−9.05 ± 0.03

* The experimental binding free energy was derived from the IC_50_ of 0.018 μM [41] and was calculated by the equation ΔG_bind_ = RTlnIC_50_.

**Table S2:** The ΔG_bind_ value (kcal/mol) in each run and the averaged ΔG_bind_ of the two focused drug candidates and BIRB796 in complex with p38α MAPK. The calculations in three independent runs of each complex showed the similar range of ΔG_bind_ value, indicating the reproducibility of end-point SIE prediction of the binding affinity. We noted that the ΔG_bind_ listed in Table 1 were from the first run since we kept them consistent with other eight remaining drug candidates.

Compounds	Run	Energy Components (kcal/mol)				
		E_vdW_	E_coul_	ΔG_RF_	ΔG_cavity_	ΔG_bind_
BIRB796	1	−78.53 ± 0.29	−9.93 ± 0.15	15.60 ± 0.20	−13.63 ± 0.04	−11.95 ± 0.04
	2	−82.37 ± 0.28	−10.85 ± 0.14	15.07 ± 0.20	−13.91 ± 0.04	−11.64 ± 0.03
	3	−82.01 ± 0.32	−8.32 ± 0.16	11.41 ± 0.19	−13.58 ± 0.04	−11.69 ± 0.04
	*Average*					**−11.76 ± 0.04**
Lomitapide	1	−77.77 ± 0.39	−5.95 ± 0.18	17.01 ± 0.24	−14.43 ± 0.04	−11.39 ± 0.05
	2	−78.41 ± 0.34	−13.12 ± 0.19	29.61 ± 0.35	−14.58 ± 0.06	−10.90 ± 0.04
	3	−81.79 ± 0.34	−6.11 ± 0.18	16.21 ± 0.24	−13.16 ± 0.04	−11.78 ± 0.04
	*Average*					**−11.35 ± 0.04**
Nilotinib	1	−71.93 ± 0.26	−12.36 ± 0.19	17.15 ± 0.17	−12.81 ± 0.04	−11.27 ± 0.03
	2	−65.37 ± 0.35	−13.35 ± 0.24	17.40 ± 0.19	−12.36 ± 0.05	−10.61 ± 0.04
	3	−69.27 ± 0.32	−12.30 ± 0.21	20.18 ± 0.18	−12.95 ± 0.04	−10.68 ± 0.04
	*Average*					**−10.85 ± 0.04**
