# Supplementary material for: Integration of In Silico Strategies for Drug Repositioning towards P38α Mitogen-Activated Protein Kinase (MAPK) at the Allosteric Site
Source: Pharmaceutics. 2022 Jul 13;14(7):1461. doi: 10.3390/pharmaceutics14071461 (PMC9321129; doi:10.3390/pharmaceutics14071461)
Supplement: Supplementary file 1 [file pharmaceutics-14-01461-s001.zip › pharmaceutics-1750839-supplementary.pdf]

pharmaceutics

MDPI

Supplementary Materials: Integration of In Silico Strategies for Drug Repositioning towards P38α Mitogen-Activated Protein Kinase (MAPK) at the Allosteric Site

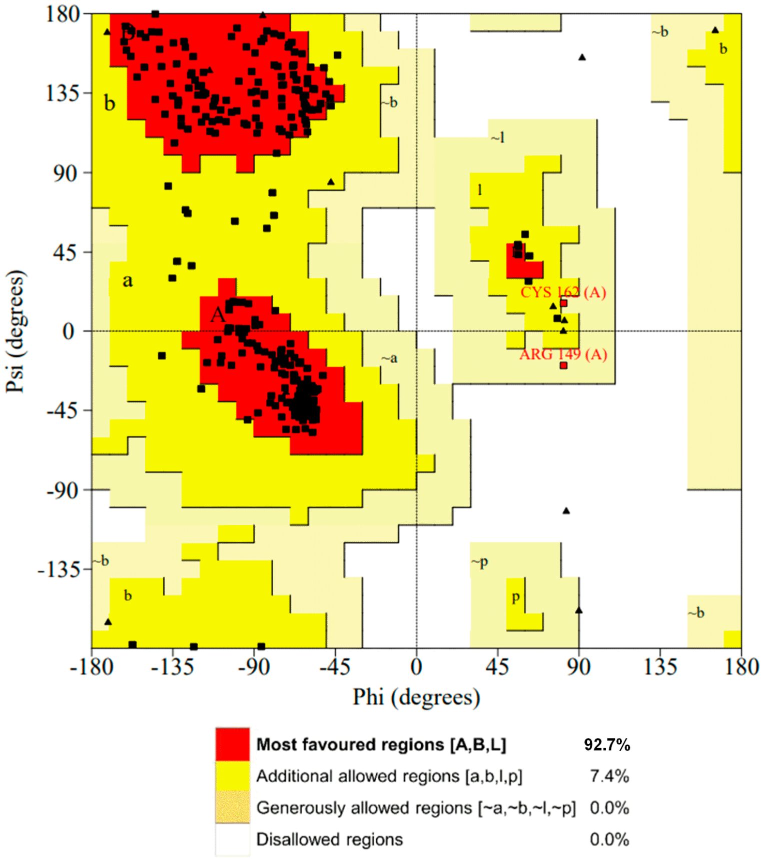

Figure S1: Ramanchandran plot analysis of a homologically constructed protein structure where the torsional conformation of amino acids was plotted. Most of them were fallen into favoured/allowed regions, which signify an acceptable quality of constructed model.

 https://doi.org/10.3390/pharmaceutics14071461

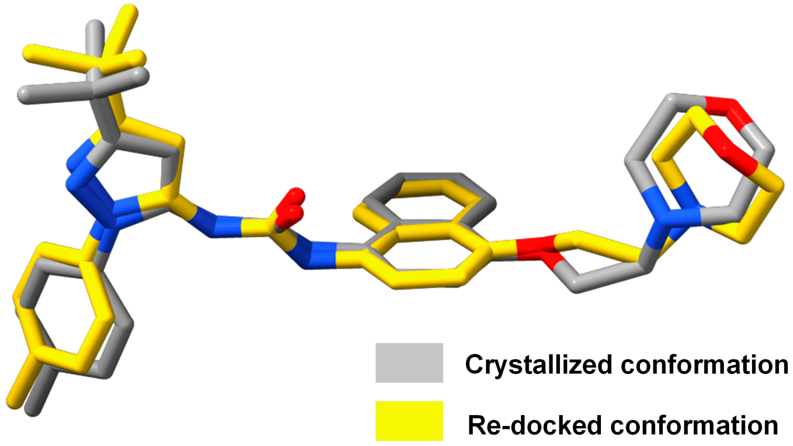

Figure S2: Alignment of the re-docked pose and available crystallized ligand (BIRB796) of p38*α* MAPK indicating a verified docking protocol used in this study.

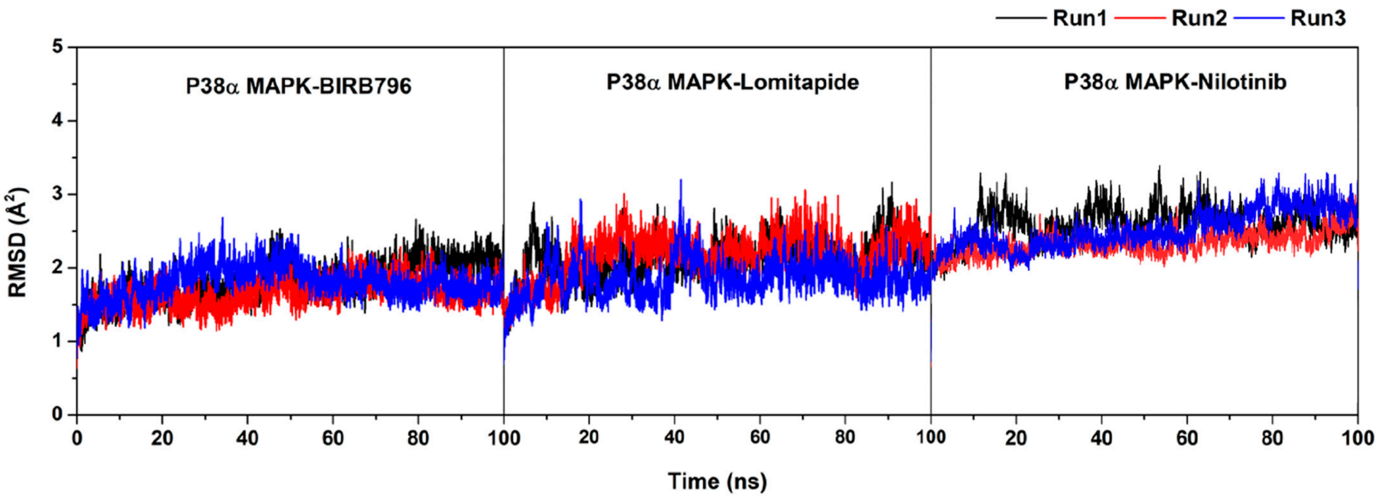

Figure S3: Plot of root-mean-square displacement (RMSD) for the protein-ligand complexes. The data were illustrated in three independent runs with different initial velocities.

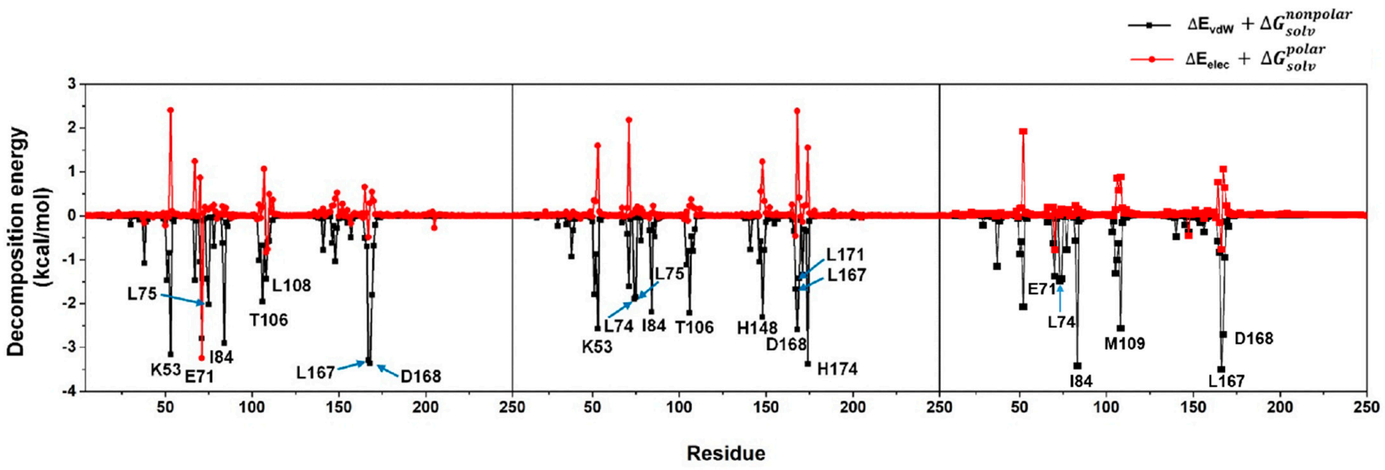

Figure S4: Analysis of per-residue VdW and electrostatic decomposition energy in which the amino acids largely contributed via VdW and electrostatic interaction energies were labelled as a one-letter code format. We noted that even though some residues (e.g., K53, H148) were noticeably stabilized through vdW interactions, they did not play a role in the binding process since they were destabilized by electrostatic charge-charge repulsion (as observed in a positive electrostatic energy). Hence, these kinds of residues were not shown in the article Figure 6.

Table S1: Summary of key pharmacophore features of BIRB796, lomitapide, and nilotinib detected by using the PharmaGist web interface.

Compounds	Total Features	Aromatic moiety	Hydrophobic moiety	H-bond donor	H-bond acceptor
BIRB796	20	4	8	2	5
Lomitapide	11	4	2	2	3
Nilotinib	16	5	3	4	4

Table S2: The ΔG_{bind} value (kcal/mol) in each run and the averaged ΔG_{bind} of the two focused drug candidates and BIRB796 in complex with p38*α* MAPK. The calculations in three independent runs of each complex showed the similar range of ΔG_{bind} value, indicating the reproducibility of end-point SIE prediction of the binding affinity. We noted that the ΔG_{bind} listed in Table 1 were from the first run since we kept them consistent with other eight remaining drug candidates.

**Compounds**	**Run**	**Energy Components (kcal/mol)**				
		E_{vdW}	E_{coul}	ΔG_{RF}	ΔG_{cavity}	ΔG_{bind}
BIRB796	1	−78.53 ± 0.29	−9.93 ± 0.15	15.60 ± 0.20	−13.63 ± 0.04	−11.95 ± 0.04
	2	−82.37 ± 0.28	−10.85 ± 0.14	15.07 ± 0.20	−13.91 ± 0.04	−11.64 ± 0.03
	3	−82.01 ± 0.32	−8.32 ± 0.16	11.41 ± 0.19	−13.58 ± 0.04	−11.69 ± 0.04
	Average					**−11.76 ± 0.04**
Lomitapide	1	−77.77 ± 0.39	−5.95 ± 0.18	17.01 ± 0.24	−14.43 ± 0.04	−11.39 ± 0.05
	2	−78.41 ± 0.34	−13.12 ± 0.19	29.61 ± 0.35	−14.58 ± 0.06	−10.90 ± 0.04
	3	−81.79 ± 0.34	−6.11 ± 0.18	16.21 ± 0.24	−13.16 ± 0.04	−11.78 ± 0.04
	Average					**−11.35 ± 0.04**
Nilotinib	1	−71.93 ± 0.26	−12.36 ± 0.19	17.15 ± 0.17	−12.81 ± 0.04	−11.27 ± 0.03
	2	−65.37 ± 0.35	−13.35 ± 0.24	17.40 ± 0.19	−12.36 ± 0.05	−10.61 ± 0.04
	3	−69.27 ± 0.32	−12.30 ± 0.21	20.18 ± 0.18	−12.95 ± 0.04	−10.68 ± 0.04
	Average					**−10.85 ± 0.04**
